# Draft Genome Sequence of Scheffersomyces spartinae ARV011, a Marine Yeast Isolate

**DOI:** 10.1128/MRA.00652-21

**Published:** 2021-11-11

**Authors:** Alex R. Villarreal, Danielle E. Campbell, Shanice S. Webster, Ryan C. Hunter

**Affiliations:** a Department of Microbiology and Immunology, University of Minnesota, Minneapolis, Minnesota, USA; b Department of Medicine, Division of Infectious Diseases, Washington University School of Medicine, St. Louis, Missouri, USA; c Edison Family Center for Genome Sciences and Systems Biology, Washington University School of Medicine, St. Louis, Missouri, USA; d Department of Microbiology and Immunology, Geisel School of Medicine at Dartmouth, Hanover, New Hampshire, USA; University of California, Riverside

## Abstract

We report the draft genome sequence of Scheffersomyces spartinae ARV011, which was isolated from the Great Sippewissett Marsh in Falmouth, Massachusetts. Sequencing was performed using the Illumina NovaSeq 6000 platform, yielding 7,598,030 read pairs 250 bp in length. This resulted in a total draft genome size of 12,132,557 bp.

## ANNOUNCEMENT

Previously assigned to the genus *Pichia* on the basis of phenotypic traits such as the ability to form ascospores, the species *spartinae* was assigned to its current genus *Scheffersomyces* in 2010 after large-subunit (LSU) rRNA (D1/D2) and small-subunit (SSU) rRNA gene sequencing provided support for its reassignment (1). Assignment of Scheffersomyces spartinae to its current genus remains controversial because of poor bootstrap support and, unlike other members of the genus, its inability to efficiently ferment d-xylose to ethanol (1, 2). However, S. spartinae is known to produce coenzyme Q9, fostering interest in the species for its potential biotechnological applications (1). The lack of sequenced S. spartinae genomes has limited progress in this area.

**FIG 1:**
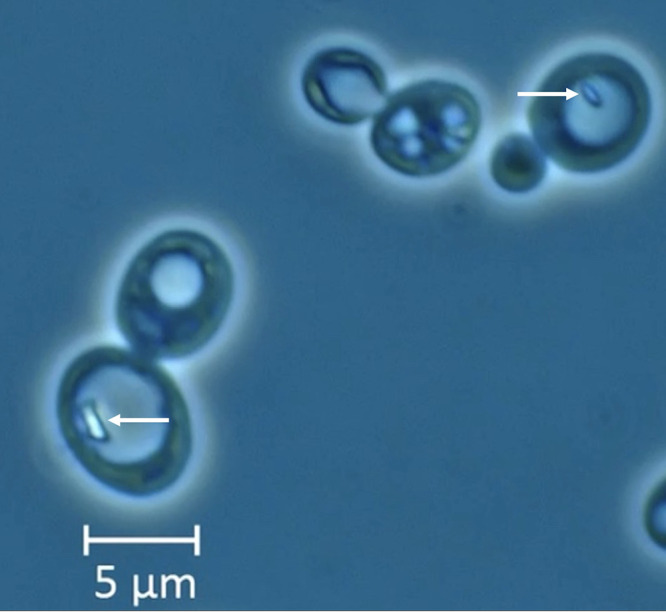
Bright-field microscopy of isolate ARV011 at ×1,000 magnification. Yeast isolate cells ranged from 4 to 10 µm in size, while the intracellular granules (white arrows) within enlarged vacuoles ranged from 1 to 2 µm in size.

S. spartinae ARV011 was isolated from marine water and sediment collected from the Great Sippewissett Marsh in Falmouth, Massachusetts. The sample was composed of brackish water, sediment, plant matter, and macroscopic photosynthetic microbial aggregates known as “pink berries” (3). Microbial enrichment from this sediment was performed using yeast extract-peptone-dextrose (YPD) broth containing antibiotics (11 µg/ml carbenicillin, 25 µg/ml chloramphenicol, and 10 µg/ml tetracycline), with incubation at 30°C for 48 h prior to culture on YPD agar. An isolate of interest, based on the microscopic appearance of enlarged vacuoles containing fast moving volutin or “dancing bodies” (4) (Fig. 1), was selected for further characterization. Following overnight culture in YPD medium, genomic DNA was extracted using the Quick-DNA bacterial/fungal miniprep kit (Zymogen). Initial identification of the isolate as S. spartinae was performed by Sanger sequencing of the internal transcribed spacer (ITS) region using the primer set ITS1-30F/ITS1-217R (5) and analysis via BLASTn against the standard nonredundant nucleotide database (6). Both the forward and reverse sequences best matched the same S. spartinae ITS and ribosomal subunit sequence (GenBank accession number KY105354.1), with the forward and reverse sequences exhibiting 98.94% and 99.20% identity, respectively. The same DNA extract was used to prepare a paired-end Illumina library using the TruSeq DNA PCR-free kit. Sequencing was performed using the Illumina NovaSeq 6000 platform, yielding 7,598,030 read pairs. Quality control was completed using KneadData v0.7.6 (https://github.com/biobakery/kneaddata), a wrapper for FastQC (7), Trimmomatic (8), and Bowtie2 (9), all with default settings. After quality control, 6,975,482 high-quality read pairs remained.

Adapter-free and quality-filtered sequences were assembled *de novo* using the SPAdes genome assembler v3.10.0 with default settings and no additional pipeline options (10). The resulting assembly graph was visualized via Bandage v0.8.1 (11) using default settings, which resulted in a total of 646 contigs (nodes), 858 connections between those contigs (edges), 19 dead ends, an *N*_50_ value of 375,049 bp, a GC content of 38.39%, and a total length of 12,201,803 bp, including overlaps. Annotation through NCBI estimated a draft genome size of 12,132,557 bp, with 103 contigs and 165 scaffolds. BLASTn was used via Bandage to reconfirm the isolate identification as S. spartinae, finding a 100% identity match to the published S. spartinae ATCC 18866 (12) partial 18S rRNA gene (GenBank accession number HQ876044.1) and a 100% match to the D1D2 region of the 26S rRNA gene (GenBank accession number HQ876052.1). Gene prediction and functional annotation were performed using default settings with Funannotate v1.8.1, a software package for gene prediction, annotation, and comparison of small eukaryotic genomes (13). Through Funannotate, Augustus, Augustus HiQ, GlimmerHMM, and snap identified a total of 14,401 genes. Also through Funannotate, Diamond mapped 550,947 proteins to the isolate genome, and 2,014 final alignments were made via Exonerate. Default databases were used for identification of genes and proteins. The full list of databases used and their version numbers are associated with the default settings of Funannotate v1.8.1. Functional annotation completeness was estimated to be above 89.6% using BUSCO v3 (14) run in protein mode and 91.3% run in genome mode with the Ascomycota lineage and Saccharomyces cerevisiae species as references. These data suggest that a nearly complete genome sequence was obtained for Scheffersomyces spartinae ARV011, which serves as a valuable resource for future studies of this organism.

### Data availability.

This whole-genome shotgun project for Scheffersomyces spartinae ARV011 has been deposited in DDBJ/ENA/GenBank under accession number JAHMUF000000000. The version described in this paper is version JAHMUF010000000. The draft genome assembly and annotation can be found under BioProject number PRJNA738152 and BioSample number SAMN19714210.
